# Supplementary material for: Multi‐dataset identification of innovative feature genes and molecular mechanisms in keratoconus
Source: J Cell Mol Med. 2024 Sep 19;28(18):e70079. doi: 10.1111/jcmm.70079 (PMC11412914; doi:10.1111/jcmm.70079)
Supplement: Supplementary file 2 — Material S2. [file JCMM-28-e70079-s002.zip › 7.GSEA analysis/1.ARL11/KEGG_VEGF_SIGNALING_PATHWAY.html]

Details for gene set KEGG\_VEGF\_SIGNALING\_PATHWAY[GSEA]

|  || Dataset | ARL11.group.cls#h\_versus\_l |
| Phenotype | group.cls#h\_versus\_l |
| Upregulated in class | l |
| GeneSet | KEGG\_VEGF\_SIGNALING\_PATHWAY |
| Enrichment Score (ES) | -0.24883385 |
| Normalized Enrichment Score (NES) | -0.93587327 |
| Nominal p-value | 0.5618861 |
| FDR q-value | 1.0 |
| FWER p-Value | 1.0 |
Table: GSEA Results Summary

  

Fig 1: Enrichment plot: KEGG\_VEGF\_SIGNALING\_PATHWAY      
 Profile of the Running ES Score & Positions of GeneSet Members on the Rank Ordered List

  

| SYMBOL | TITLE | RANK IN GENE LIST | RANK METRIC SCORE | RUNNING ES | CORE ENRICHMENT || 1 | PIK3R3 | na | 2180 | 0.284 | -0.0206 | No |
| 2 | PIK3CG | na | 2870 | 0.247 | -0.0037 | No |
| 3 | NOS3 | na | 4290 | 0.190 | -0.0163 | No |
| 4 | PLCG1 | na | 4334 | 0.189 | 0.0107 | No |
| 5 | CHP2 | na | 4525 | 0.182 | 0.0323 | No |
| 6 | RAC3 | na | 4612 | 0.179 | 0.0565 | No |
| 7 | PLA2G4E | na | 4751 | 0.174 | 0.0784 | No |
| 8 | PIK3R1 | na | 6079 | 0.135 | 0.0603 | No |
| 9 | PLCG2 | na | 6096 | 0.135 | 0.0799 | No |
| 10 | PLA2G3 | na | 6310 | 0.129 | 0.0930 | No |
| 11 | PIK3R2 | na | 8783 | 0.078 | 0.0333 | No |
| 12 | PLA2G12B | na | 9215 | 0.072 | 0.0316 | No |
| 13 | AKT2 | na | 9348 | 0.070 | 0.0381 | No |
| 14 | PIK3R5 | na | 10118 | 0.059 | 0.0247 | No |
| 15 | PRKCA | na | 10617 | 0.052 | 0.0181 | No |
| 16 | KRAS | na | 10630 | 0.052 | 0.0256 | No |
| 17 | MAPK11 | na | 11276 | 0.044 | 0.0136 | No |
| 18 | SPHK1 | na | 12671 | 0.030 | -0.0222 | No |
| 19 | NFATC3 | na | 12823 | 0.028 | -0.0223 | No |
| 20 | MAPK12 | na | 13078 | 0.026 | -0.0257 | No |
| 21 | NFATC2 | na | 13211 | 0.025 | -0.0258 | No |
| 22 | PLA2G2C | na | 13424 | 0.024 | -0.0284 | No |
| 23 | PIK3CA | na | 13512 | 0.023 | -0.0275 | No |
| 24 | JMJD7-PLA2G4B | na | 14509 | 0.015 | -0.0540 | No |
| 25 | HSPB1 | na | 14853 | 0.012 | -0.0621 | No |
| 26 | KDR | na | 15337 | 0.009 | -0.0747 | No |
| 27 | MAPKAPK3 | na | 15664 | 0.006 | -0.0832 | No |
| 28 | RAC1 | na | 16390 | 0.001 | -0.1040 | No |
| 29 | MAP2K2 | na | 16997 | -0.003 | -0.1210 | No |
| 30 | PLA2G4A | na | 17245 | -0.005 | -0.1274 | No |
| 31 | PRKCG | na | 17545 | -0.007 | -0.1349 | No |
| 32 | MAPK14 | na | 17567 | -0.007 | -0.1345 | No |
| 33 | PLA2G6 | na | 17692 | -0.008 | -0.1368 | No |
| 34 | PLA2G12A | na | 17822 | -0.009 | -0.1392 | No |
| 35 | AKT1 | na | 18085 | -0.011 | -0.1451 | No |
| 36 | CDC42 | na | 18361 | -0.013 | -0.1512 | No |
| 37 | HRAS | na | 18435 | -0.013 | -0.1513 | No |
| 38 | SH2D2A | na | 18599 | -0.014 | -0.1539 | No |
| 39 | RAC2 | na | 18868 | -0.016 | -0.1592 | No |
| 40 | PPP3R1 | na | 19029 | -0.017 | -0.1613 | No |
| 41 | BAD | na | 19342 | -0.019 | -0.1674 | No |
| 42 | PPP3CA | na | 19531 | -0.021 | -0.1697 | No |
| 43 | PLA2G2E | na | 19951 | -0.023 | -0.1783 | No |
| 44 | CASP9 | na | 20358 | -0.026 | -0.1861 | No |
| 45 | MAPK13 | na | 20970 | -0.031 | -0.1991 | No |
| 46 | CHP1 | na | 21036 | -0.032 | -0.1963 | No |
| 47 | PPP3CB | na | 21103 | -0.032 | -0.1934 | No |
| 48 | PTK2 | na | 22303 | -0.043 | -0.2216 | No |
| 49 | MAPK3 | na | 22614 | -0.046 | -0.2236 | No |
| 50 | RAF1 | na | 23336 | -0.054 | -0.2364 | No |
| 51 | NFATC4 | na | 23442 | -0.055 | -0.2312 | No |
| 52 | PLA2G10 | na | 23788 | -0.060 | -0.2323 | No |
| 53 | PIK3CB | na | 23818 | -0.060 | -0.2242 | No |
| 54 | SRC | na | 24048 | -0.063 | -0.2214 | No |
| 55 | MAPK1 | na | 24626 | -0.071 | -0.2274 | No |
| 56 | NFAT5 | na | 25369 | -0.081 | -0.2368 | Yes |
| 57 | PIK3CD | na | 25759 | -0.087 | -0.2351 | Yes |
| 58 | MAPKAPK2 | na | 25894 | -0.089 | -0.2257 | Yes |
| 59 | PXN | na | 26293 | -0.095 | -0.2231 | Yes |
| 60 | SPHK2 | na | 26389 | -0.096 | -0.2114 | Yes |
| 61 | PPP3R2 | na | 26430 | -0.097 | -0.1981 | Yes |
| 62 | PTGS2 | na | 26519 | -0.099 | -0.1859 | Yes |
| 63 | AKT3 | na | 27547 | -0.120 | -0.1976 | Yes |
| 64 | NRAS | na | 27790 | -0.126 | -0.1859 | Yes |
| 65 | PPP3CC | na | 27901 | -0.128 | -0.1699 | Yes |
| 66 | PLA2G2D | na | 27985 | -0.130 | -0.1528 | Yes |
| 67 | VEGFA | na | 28646 | -0.148 | -0.1499 | Yes |
| 68 | SHC2 | na | 29625 | -0.176 | -0.1518 | Yes |
| 69 | PLA2G2F | na | 30623 | -0.208 | -0.1495 | Yes |
| 70 | MAP2K1 | na | 31070 | -0.223 | -0.1291 | Yes |
| 71 | PLA2G5 | na | 32093 | -0.264 | -0.1193 | Yes |
| 72 | NFATC1 | na | 32265 | -0.273 | -0.0835 | Yes |
| 73 | PLA2G2A | na | 32310 | -0.275 | -0.0438 | Yes |
| 74 | PLA2G1B | na | 33526 | -0.362 | -0.0248 | Yes |
| 75 | PRKCB | na | 33863 | -0.399 | 0.0250 | Yes |
Table: GSEA details [plain text format]

  

Fig 2: KEGG\_VEGF\_SIGNALING\_PATHWAY      
 Blue-Pink O' Gram in the Space of the Analyzed GeneSet

  

Fig 3: KEGG\_VEGF\_SIGNALING\_PATHWAY: Random ES distribution      
 Gene set null distribution of ES for **KEGG\_VEGF\_SIGNALING\_PATHWAY**
